# Insights into the Risk Factors and Outcomes of Post-COVID-19 Syndrome—Results from a Retrospective, Cross-Sectional Study in Romania

**DOI:** 10.3390/life14111519

**Published:** 2024-11-20

**Authors:** Ioana Bejan, Corneliu Petru Popescu, Simona Maria Ruta

**Affiliations:** 1 Faculty of Medicine, Carol Davila University of Medicine and Pharmacy, 020956 Bucharest, Romania; ioana.bejan@rez.umfcd.ro (I.B.); corneliu.popescu@umfcd.ro (C.P.P.); 2 Victor Babes Hospital for Infectious and Tropical Diseases, 030303 Bucharest, Romania; 3 Stefan S Nicolau Institute of Virology, 030304 Bucharest, Romania

**Keywords:** post-COVID-19 syndrome, long COVID, COVID-19 sequelae, SARS-CoV-2 vaccination

## Abstract

Post-Coronavirus Disease 2019 (post-COVID-19) syndrome represents a cluster of persistent symptoms following Severe Acute Respiratory Syndrome Coronavirus 2 (SARS-CoV-2) infection that can severely affect quality of life. The pathogenic mechanisms and epidemiology in different regions are still under evaluation. To assess the outcomes of post-COVID-19 syndrome, we performed a questionnaire-based, cross-sectional study in previously infected individuals. Out of 549 respondents, (male:female ratio: 0.32), 29.5% had persistent symptoms at 3 months, 23.5% had persistent symptoms at 6 months, and 18.3% had persistent symptoms at 12 months after the initial infection. The most common symptoms included fatigue (8.7%), sleep disturbances (7.1%), and cognitive impairment (6.4%). The risk of developing post-COVID-19 syndrome increased for those with more symptoms in the acute phase (OR 4.24, *p* < 0.001) and those experiencing reinfections (OR 2.405, *p* < 0.001), while SARS-CoV-2 vaccination halved the risk (OR = 0.489, *p* = 0.004). Individuals with post-COVID-19 syndrome had a 5.7-fold higher risk of being diagnosed with a new chronic condition, with 44% reporting cardiovascular disease, and a 6.8-fold higher likelihood of needing medical care or leave. Affected individuals reported significant impairments in mobility, pain/discomfort, and anxiety/depression, with 20.7% needing to adjust their work schedules. Overall, patients with post-COVID-19 syndrome require ongoing monitoring and rehabilitation, and further socio-economic impact studies are needed.

## 1. Introduction

Emerging evidence increasingly supports the existence of long-term sequelae following SARS-CoV-2 infection, even among individuals who experienced asymptomatic or mild forms of the acute disease. According to an international Delphi consensus [1], post-COVID-19 syndrome is characterized by the persistence of symptoms or the emergence of new symptoms at least three months post infection, following the exclusion of other potential diagnoses. These symptoms must last for a minimum of two months and significantly impair the individual’s quality of life [1,2].

A meta-analysis conducted on post-infection outcomes indicates that, two years after initial infection, the most commonly reported symptoms include fatigue (12–47%), cognitive impairment (12.6–45.8%), and pain (4.9–12.8%), in addition to psychological distress manifesting as anxiety or depressive episodes [3]. The clinical presentation of post-COVID-19 syndrome remains challenging to quantify and delineate due to its overlap with various post-viral pathologies, such as myalgic encephalomyelitis/chronic fatigue syndrome (ME/CFS) and postural orthostatic tachycardia syndrome (POTS) [4,5].

Four years post-emergence of SARS-CoV-2, the reported prevalence of post-COVID-19 syndrome is estimated to range from 1% to 10% among non-hospitalized patients, while it escalates to 50–70% among those who were hospitalized during the acute phase, particularly affecting individuals aged 36–50 years [6]. The etiology of this syndrome remains poorly understood, with multiple contributing factors identified, including persistent systemic inflammation, immune dysregulation characterized by the depletion of SARS-CoV-2-specific CD8+ T cells, and a lack of coordination between cellular and humoral immune responses [7]. Additionally, dysregulation of the complement system and increased activation—potentially triggered by the reactivation of latent herpesviruses, especially Epstein–Barr virus—have also been implicated [8]. Other factors may involve potential reservoirs of SARS-CoV-2 in tissues, alterations in the microbiota, or autoimmune mechanisms [4].

Despite advances in rehabilitation support for patients in developed countries, a global deficit in understanding, awareness, and information regarding post-COVID-19 syndrome persists. This underscores the urgent need for large-scale clinical trials aimed at establishing therapeutic protocols to enhance the quality of life of affected individuals [4]. In Romania, 3.52 million cases of confirmed SARS-CoV-2 infections were reported from February 2020 to April 2024. There were 68.934 deaths, out of which 86.6% were recorded in persons older than 60 years of age and 93.8% were recorded in persons with associated comorbidities. COVID-19 vaccination in Romania started on 27 December 2020 to the end of 2023; more than 8 million people have been vaccinated, representing 43% of the population, a lower percentage compared to Western European countries. The vaccination rate among healthcare workers was almost double compared to the vaccination rate among the general population [9]. The majority of studies addressing post-COVID-19 syndrome are limited to case series or cross-sectional analyses conducted in single medical centers or with small patient cohorts [10,11,12]. Consequently, there is a notable lack of comprehensive monitoring for individuals suffering from post-COVID-19 sequelae, alongside an insufficient assessment of their socio-medical needs. This study aims to elucidate the outcomes associated with post-COVID-19 syndrome in the Romanian population and to identify potential risk factors influencing its development and progression.

## 2. Materials and Methods

### 2.1. Study Participants

In this cross-sectional, observational study, an online questionnaire was distributed between 6 March and 15 May 2023, both to former patients admitted to Victor Babes Clinical Hospital of Bucharest with COVID-19 and on social media groups built around medicine-related issues. The inclusion criteria for the selected subjects were defined as being at least 18 years old and having a positive history of SARS-CoV-2 infection. Subjects were informed about the policies of processing personal data and informed consent was obtained before proceeding to the questionnaire—Supplementary Materials. The study was approved by the IRB committees of the Victor Babes Clinical Hospital of Bucharest.

### 2.2. Sample Size

To calculate the adequate sample size of the studied cohort, an online tool [13] was accessed, with a confidence interval set at 95% in an expected population portion of 30%, resulting in a minimum number of 323 subjects enrolled in the study for a proper strength of associations.

### 2.3. Questionnaire

The questionnaire consisted of 36 items, grouped into multiple sections, which included (1) the general information of the participants, (2) general health and (3) quality of life pre-infection, (4) SARS-CoV-2 vaccination status, (5) SARS-CoV-2 infection, (6) post-COVID-19 symptoms, (7) quality of life after infection. Post-COVID-19 symptoms were evaluated by choosing from a list of 16 symptoms, those present in the first 4 weeks and then those persisting at 3, 6, and 12 months after the initial infection. Aside from the listed symptoms, the patients had an open-answer type question where they could write down any other unlisted symptom they had been afflicted with during and after the acute SARS-CoV-2 infection. To assess quality of life pre and post infection, an adapted version of the EuroQoL 5D-3L scale [14] was used, with categories including self-care, mobility, activities of daily living, chronic pain, and depression/anxiety. Subjects attributed scores from 1 (complete independence and lack of discomfort) to 3 (severe problems in that specific category).

### 2.4. Data Analysis

After the initial manual sorting of data, the visual instruments available on the Google Forms platform, as well as Microsoft Excel 2013 and IBM SPSS v.20, were used to perform the descriptive analysis and analytical associations of the results with age, sex, educational level, urban/rural background, occupational status, health history, quality of life, acute and chronic clinical manifestations of SARS-CoV-2 infection. Respondents were then separated using the definition for post-COVID-19 syndrome given by the international Delphi Consensus [1] into two main subgroups: those afflicted with post-COVID-19 syndrome and those with complete recovery who returned to their full initial health after the acute infection. Given the expected non-gaussian distribution of the results, mainly non-parametric tests were deployed to check for statistically relevant associations, which could aid in delineating the epidemiological and clinical aspects within the subgroup of subjects suffering from post-COVID-19 syndrome, with a minimum standard threshold of significance set at *p*-value < 0.05.

## 3. Results

### 3.1. Characteristics of the Enrolled Subjects

Out of 607 people who accessed the questionnaire, 58 declared never having been infected with SARS-CoV-2, thus being ineligible for participation, the final analyzed cohort consisted of 549 subjects. The general characteristics of the included subjects are summarized in Table 1.

### 3.2. SARS-CoV-2 Vaccination Status

Out of all respondents, 43.1% received a complete SARS-CoV-2 vaccination scheme (with 2 initial doses and a booster at least 10 months after), and 32.4% received only 2 vaccine doses (Table 2). The rest (23.6%) were unvaccinated or received only one vaccine dose. Most of the respondents received an mRNA vaccine, mainly Comirnaty/Pfizer (67.4%), but also SpikeVax/Moderna (5.6%), while adenoviral vector-based vaccines such as Vaxzevria/Astra Zeneca or Jcovden/Jansen were administered in 10.2% of the respondents. Out of the total number of subjects, 53.1% were infected after vaccination, out of which 22.8% were infected after having a booster dose administered (with a median of 212 days between the last vaccine dose and infection).

### 3.3. Diagnosis of SARS-CoV-2 Infection

**Figure 1:**
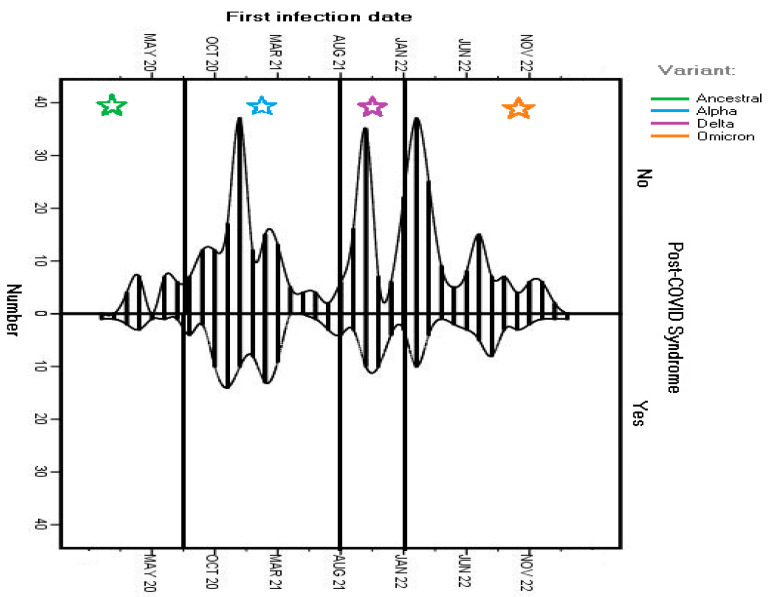
SARS-CoV-2 infections reported in the study cohort throughout the pandemic and the dominant viral variant at that time in Romania.

Most patients (88.5%) had a laboratory-confirmed SARS-CoV-2 infection (via Rt-PCR or rapid antigen testing) with mild symptomatology. The infections were equally distributed across the pandemic period, with small peaks in the winter seasons: 77 cases (14%) were reported between November and December 2020, 81 cases (14.7%) were reported between October and December 2021, and 101 cases (18.4%) were reported between January and March 2022, with them visually depicted in a bar-type graph in order to analyze possible relations with the dominant viral variant at that time in Romania (Figure 1).

A third of the respondents had experienced at least one reinfection, with similar or worse symptomatology compared to the first infection in almost half of the cases (Table 3).

There were no significant differences between those hospitalized and non-hospitalized according to gender (*p* = 0.633), smoking status (*p* = 0.58), or lengths between vaccine and primary infection (*p* = 0.234). Hospitalized patients were older compared to those who were not hospitalized (age group: (in the age group 45–54 years vs. 35–44 years, *p* < 0.001)*,* obese (OR 2.72 [95% CI 1.45–5.09], *p* = 0.001), and had reinfections (OR-1.83 [95% CI 1.08–3.1], *p* = 0.023). Only 24 (38%) of the hospitalized patients reported having received one of the antivirals available in Romania at the time of admission.

### 3.4. Persistence of Symptoms After the Acute Infection

Four weeks after their first infection, 301 (54.8%) of the enrolled subjects completely recovered, while 157 (28.6%) still had persistent symptoms and 91 (16.6%) considered themselves not fully recovered but could not point out any particular remaining symptom. Out of those who denied complete recovery after 4 weeks, 158 (63.7%) declared that their overall health had been improving over time, 38 (15.3%) declared a cyclicality of their symptomatology, 20 (8%) said that their symptoms continued with varying intensity across time, and only 21 (8.5%) declared either a constant intensity or worsening of their symptomatology.

Out of the total enrolled subjects, 162 (29.5%) had persistent symptoms at 3 months post infection, with them being classified as having post-COVID-19 syndrome. The most frequently affected were women in the 35–44 age group (44/162 subjects—27.2% of the post-COVID-19 patients). Overall, only 33 men (24.6%) had symptoms of long COVID compared to 129 women (31.2%).

The number of patients with persistent symptoms decreased over time: 129 (23.5%) at 6 months post-infection and 101 (18.3%) at 12 months after infection. The most prevalent persisting symptoms are listed in Table 4. At 12 months post-SARS-CoV-2 infection, all symptoms were less prevalent, with higher frequency maintained only for fatigue, sleep disturbances, cognitive dysfunctions, anxiety/depression, and palpitations.

### 3.5. Perceived Aggravating Factors for Post-COVID-19 Syndrome

Asked about perceived aggravating factors of their post-COVID-19 symptoms, 60.5% of the 162 subjects with persisting symptoms at 3 months indicated physical effort, 51.8% indicated a high level of stress, 32.1% indicated insufficient sleep hours, 25.9% indicated extended periods of orthostatism, 16.6% declared that going back to work played a part in aggravating their symptoms, and 15.4% recognized fluctuations dependent on the time of day.

### 3.6. Risk Factors for Post-COVID-19 Symptomatology

The vaccination status, the number of symptoms in the first 4 weeks, the need for hospitalization in the acute period and any number of reinfections were significantly associated with disease risk (Table 5). No other variables (gender, age, BMI, and smoking status) significantly influenced the likelihood of being at risk of post-COVID syndrome.

**Figure 2:**
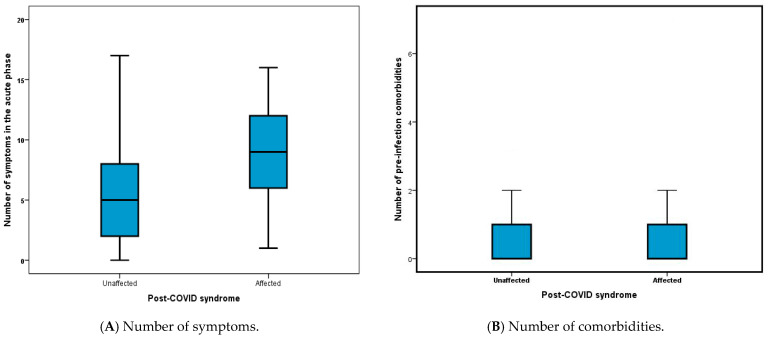

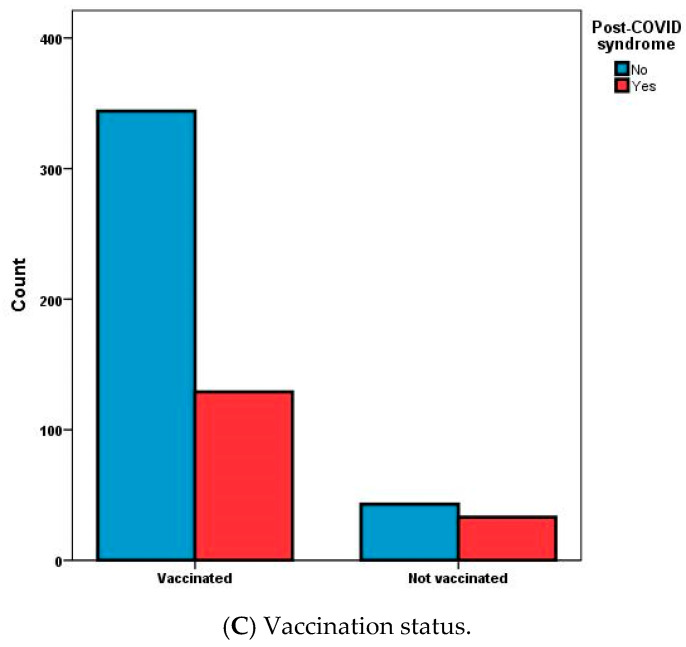
Impact of the different risk factors on the development of post-COVID-19 syndrome. (**A**) number of symptoms during the acute phase of the infection, (**B**) number of pre-infection comorbidities, (**C**) vaccination status.

A box plot graph (Figure 2) is used as a visual depiction of the different risk factors between those who later developed post-COVID-19 syndrome and those unaffected. Several symptoms during the acute phase were highly predictable for the development of post-COVID-19 symptoms (dyspnea, fatigue, cognitive dysfunction, sleep disturbances), with the highest risk identified among those presenting with anxiety or depression during the acute illness, increasing the likelihood of post-COVID-19 syndrome by more than five-fold (OR 5.373; 95% CI 3.56–8.10).

### 3.7. Effect of SARS-CoV-2 Vaccination on the Development and Evolution of Post-COVID-19 Symptomatology

Vaccination was a protective factor for the development of long COVID syndrome, almost halving the risk (OR = 0.489 [95% CI 0.29–0.8], *p* = 0.004). The protective effect is present both for those vaccinated with two doses (OR = 0.62 [95% CI 0.41–0.93], *p* = 0.023) and three doses (OR = 0.49 [95% CI 0.33–0.73], *p* < 0.001). Out of those vaccinated who still developed post-COVID-19 syndrome, the majority (100; 77.5%) cannot report a beneficial effect of vaccination on their ongoing symptoms or overall health, and only 9 (7%) reported an improvement in their persistent symptoms lasting from the infection, whilst 20 (15.5%) described an exacerbation of the symptoms after getting vaccinated.

### 3.8. Multivariate Analysis

A logistic regression and ROC curve were conducted with the statistically relevant risk factors, in order to better assess their possible use as a clinical tool in predicting those at risk of developing long COVID (Table 6).

The number of acute symptoms in the first 4 weeks described the most reliable AUC (0.716, *p* < 0.001). Moreover, given the consistent positive OR results obtained in the multivariate regression, all studied factors add their own risk of developing long COVID.

### 3.9. Health Status and Quality of Life in Subjects with Long COVID

Overall, during the first 3 months post-infection, 61 (11.2%) of patients were diagnosed with a new chronic condition. Out of these, 27 (44.2%) received a diagnosis of cardiovascular disease, 18 (29%) were diagnosed with anxiety or depression, 15 (24.6%) were diagnosed with an autoimmune disease, 9 (14.7%) were diagnosed with neurologic conditions, 6 (9.8%) were diagnosed with diabetes mellitus, and 5 (8.2%) were diagnosed with a malignancy.

Subjects with persistent symptomatology have a 5.7-fold (OR = 5.71 [95% CI 3.24–10.06], *p* < 0.001) higher risk of being diagnosed with a new chronic condition within the first 3 months after acute infection. Furthermore, the number of newly diagnosed conditions is significantly higher among those with post-COVID-19 syndrome (*p* < 0.001, Mann–Whitney U test). Likewise, there is a 6.8-fold higher probability (OR = 6.88 [95% CI 4.53–10.46], *p* < 0.001) of needing specialized medical care, hospital admission, or extension of sick/unpaid leave due to long COVID.

**Figure 3:**
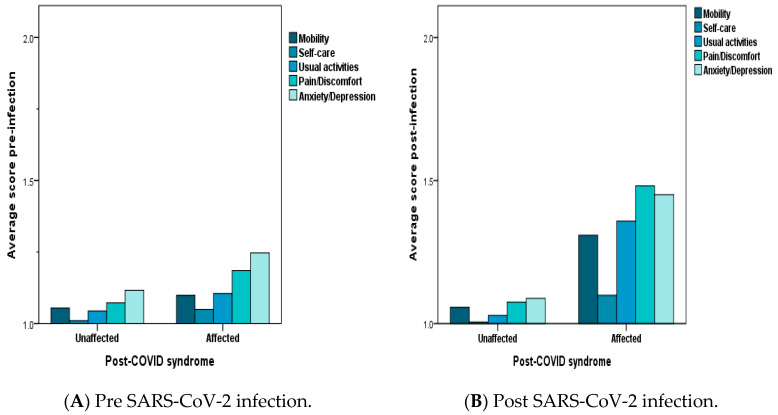
Average self-assessed health status for five areas of quality of life: Panel (**A**)—Pre-infection with SARS-CoV-2—comparison between subjects later diagnosed with long COVID and those unaffected; Panel (**B**)—Post-infection with SARS-CoV-2—comparison between subjects later diagnosed with long COVID and those unaffected, adapted from the EuroQoL 5D-3L scale [13]. Attributable scores ranged from 1—complete independence and lack of discomfort to 3—severe problems in the specified category.

According to the adapted EuroQoL 5D-3L mini-questionnaire of the perceived overall state of health [14], subjects later diagnosed with post-COVID-19 syndrome had higher scores for impairment of overall health status even in the pre-infection period (especially in terms of pain/discomfort and anxiety/depression), but there was significant impairment, especially in mobility, with increased scores for pain/discomfort and anxiety/depression after COVID-19 infection (*p* < 0.001, MWU test) (Figure 3, panels A and B).

### 3.10. Medical Resources Accessed for Post-COVID-19 Syndrome

In total, 60 participants (37%) were seen by a specialist for their post-COVID-19 symptomatology, 29 (17.9%) were seen by their general practitioner, 11 (6.8%) had multiple presentations to the emergency room without further hospitalization, 13 (8%) were admitted to a specialized rehab clinic, 9 (5.5%) had to be hospitalized for their post-COVID-19 syndrome, and 8 (4.9%) reported taking extended medical leave from work. Most of the enrolled subjects, 427 (77.7%), had returned to work with the same schedule and activities as before infection. Out of 101 patients with persistent symptoms after 12 months, 9 (8.9%) had to limit their work schedule or activities, 8 (7.9%) did not return to work, and 4 (3.9%) had to change their job due to - syndrome.

## 4. Discussion

Our study indicates that a significant proportion of individuals infected with SARS-CoV-2, regardless of the viral variant, continue to experience persistent symptoms at 3-, 6-, and 12-months post infection. The prevalence of post-COVID-19 syndrome (defined as symptoms arising or persisting at least three months following the onset of infection, according to the WHO Delphi consensus [1]) is 29.5%. However, this prevalence decreased to 18.3% at 12 months, with a corresponding reduction in the overall prevalence of symptoms.

Globally, there is considerable heterogeneity in studies on post-COVID-19 syndrome, attributed to the absence of standardized questionnaires for assessing clinical outcomes and their implications. This variability introduces multiple biases and confounding factors, complicating data synthesis in meta-analyses [15]. Nonetheless, large-scale studies in 2023 estimate that between 65 and 100 million individuals worldwide are affected by long COVID, with an overall prevalence of 6.8% among non-institutionalized adults in the United States [6].

International data suggest that most patients with post-COVID-19 syndrome had non-hospitalized acute infections [16,17], a trend also reflected in our findings, where most subjects experienced mild acute infections. We did not identify an influence of the SARS-CoV-2 variant on the symptoms associated with post-COVID-19 syndrome. SARS-CoV-2 infections reported by this study’s participants were almost equally distributed during the main COVID-19 waves in Romania, with small peaks dispersed in the last months of 2020 (when the ancestor virus was still prevalent in Romania), during the last months of 2021 (in the midst of the wave caused by the Delta variant), and between January and March 2022 (when the Omicron variant became prevalent) [18,19,20]. Other studies have suggested possible distinct profiles of post-COVID-19 syndrome [21], although the increased risk associated with the Alpha and Delta variants compared to Omicron seemed attributable to the severity of the disease and intensive care admission [22].

Notably, both the number of symptoms during the acute phase and the incidence of reinfections emerged in our study as risk factors for developing post-COVID-19 syndrome. This observation aligns with prior observations indicating that higher viral replication levels may contribute to persistent symptoms in non-hospitalized patients [23]. It has also been hypothesized that long COVID may result from initial asymptomatic infections affecting multiple organs, apart from the respiratory tract, leading to a persistent inflammatory response due to elevated viremia [23,24].

Our cohort predominantly comprised females (75.2%), mainly aged 35–44 years, residing in urban areas (90.9%), and possessing high educational levels (68.7%). This demographic distribution may be influenced by the tendency for online questionnaires to attract responses from women with higher education, as observed in other long COVID studies [25]. However, it is noteworthy that many inpatient studies report a higher prevalence of long COVID among women, complicating the quantification of potential selection bias [4,26,27].

Most extensive studies on post-COVID-19 syndrome evaluate a wide array of symptoms over time [16,23,27]. After a comprehensive literature review, we compiled a list of 16 commonly reported symptoms for participants. The results indicated that the most frequently reported symptoms at 3, 6, and 12 months included fatigue, cognitive dysfunction, palpitations, and sleep disturbances. Fatigue, in particular, has been associated with decreased hemoglobin levels, potentially linked to dysregulated iron metabolism impacting erythropoiesis [28,29]. Nevertheless, fatigue is inherently subjective and also prevalent in the general population, which may introduce biases related to the social and psychological ramifications of the pandemic itself, with social restrictions and daily life disturbances.

Meanwhile, at the 12-month follow-up, the persistence of fatigue, alongside cognitive dysfunction and sleep disturbances, was notable and consistent with findings from multiple meta-analyses on long COVID [4], potentially indicating sensorimotor deficits [30]. Cognitive impairments in long COVID patients frequently co-occur with depression and functional limitations, adversely impacting full-time employment prospects [31,32].

Importantly, our study noted a general improvement in the overall health status reported by the majority of participants (63.7%) from 3 to 12 months post-infection, with only a small fraction (23.8%) experiencing a cyclical worsening of symptoms. Persistent symptoms significantly impacted quality of life, as demonstrated through an adapted EuroQoL 5D-3L scale (*p* < 0.001, MWU test). Participants reported increased limitations in mobility, alongside pain and anxiety/depression. Specific phenotypes of pain in long COVID patients often include neoplastic pain, frequently accompanied by fatigue, sleep disturbances, and emotional distress [33]. These findings underscore the necessity for an integrated psycho-socio-medical care approach to enhance quality of life and reduce disability. Recent studies show that rehabilitation and resistance training has the potential to alleviate symptoms and increase physical ability, especially in older adults [34,35].

Additionally, our study found a significantly higher prevalence of newly diagnosed chronic conditions among long COVID patients, with cardiovascular disease being the most common. Several studies have reported an increased risk of new acute cardiovascular events in post-COVID-19 patients, associated with arterial stiffness [36,37]. The increased risk of autoimmune diseases, neurological conditions, and diabetes mellitus detected in our cohort also warrants long-term monitoring. Initial studies have documented frequent hospital readmissions, the need for palliative and long-term care, and multiple visits to the emergency department among patients with post-COVID-19 syndrome [38]. Interestingly, in our cohort, less than 5% of participants reported needing to change or quit their jobs due to post-COVID-19 syndrome, a figure substantially lower than previous reports [26,32,39]. Given the relatively young age of our participants (35–45 years), potential biases associated with older age and comorbidities are minimized [15,34].

Crucially, our study demonstrates that individuals vaccinated against COVID-19—either prior to or following acute infection—exhibited a significantly reduced risk of developing COVID-19 sequelae compared to unvaccinated individuals. This adds to a growing body of evidence supporting the protective role of SARS-CoV-2 vaccination against post-COVID-19 syndrome, resulting in lower prevalence and shorter duration of persistent symptoms, even among vaccinated individuals experiencing breakthrough infections [40,41].

However, this study is subject to several limitations. The cross-sectional design does not permit real-time tracking of post-COVID-19 syndrome development following the initial infection or subsequent reinfections. Additionally, utilizing a questionnaire may introduce selection bias, favoring responses from women with higher education levels and younger adults—more than two-thirds of the entire studied population have an advanced education level, more than 90% of them come from an urban environment, and 54,4% are women aged between 25–55 years. Consequently, the population in this study may not be nationally representative, and associations drawn should be interpreted cautiously, especially considering the infrequent representation of those who were hospitalized in the ICUs with COVID-19. Reporting biases may also arise from the accuracy and reliability of self-reported data, as definitive proof of infection was available only for the previously hospital-admitted patients, and participants may struggle to recall events accurately. Standardized tools or internationally approved questionnaires available on a multicentric national level would have been necessary to objectively describe the epidemiology and clinical findings of post-COVID-19 syndrome. Moreover, the lack of data concerning reinfection date could erroneously paint the picture of the overall infection rate over time, especially regarding its relation to the viral variant waves in Romania. Finally, we have to emphasize that the statistically significant associations between several risk factors and the development of post-COVID-19 syndrome could not be considered definitive causes, as the mechanistic processes behind this syndrome are yet to be elucidated.

Despite these limitations, our study provides valuable insights into the outcomes of post-COVID-19 syndrome in the Romanian population and outlines a potential framework for future research in this domain. While a recovery protocol for post-COVID-19 sequelae was implemented in Romania in April 2021 [42], there is still a lack of national health programs dedicated to the diagnosis and treatment of affected individuals.

## 5. Conclusions

SARS-CoV-2 infection has resulted in a significant number of individuals experiencing residual symptoms and disabilities that substantially impair their quality of life and ability to function in social and professional contexts. A large-scale, multicenter national study, with a prospective design, including individuals with all forms of acute illness, would enhance our understanding of the various clusters of post-COVID-19 symptoms. This approach would allow for the identification of different risk factors, behaviors, and demographic profiles and would facilitate a prospective follow-up of patient outcomes.

## Figures and Tables

**Table 1:** General characteristics of the enrolled subjects.

Demographics	Subgroup	Number-N, (%)
Gender	Male	134 (24.4%)
	Female	413 (75.2%)
	Prefer not to disclose	2 (0.4%)
Age group	18–24	107 (19. 5%)
	25–34	116 (21.1%)
	35–44	153 (27.9%)
	45–54	124 (22.6%)
	55–64	40 (7.3%)
	65–74	8 (1.5%)
	75+	1 (0.2%)
Background	Urban	499 (90.9%)
	Rural	50 (9.1%)
Educational level	Bachelor’s degree or higher	377 (68.7%)
	Secondary form of education	161 (29.3%)
	Primary education	11 (2%)
Healthcare worker	Yes	239 (43.5%)
	No	307 (55.9%)
*Health status and habits*		
Smoking status	Never smoker	270 (49.2%)
	Current smoker	115 (20.9%)
	Occasional smoker	69 (12.6%)
	Former smoker (1 year+)	92 (16.8%)
BMI	<30	474 (86.3%)
	>30	75 (13.7%)
Pre-infection chronic condition	Hypertension	42 (7.6%)
	Other cardiovascular disease	22 (4%)
	Autoimmune/endocrine	31 (5.7%)
	Anxiety/depression	27 (4.9%)
	Chronic pulmonary disease	20 (3.6%)
	Anemia	18 (3.3%)
	Diabetes mellitus	13 (2.4%)
	Neurological condition	5 (0.9%)
	Malignancy	5 (0.9%)
	Chronic kidney disease	4 (0.7%)
	Chronic hepatic disease	3 (0.5%)
	Immune deficiency	3 (0.5%)
	Others	15 (2.7%)

BMI—body mass index.

**Table 2:** Vaccination status of the study population.

	Vaccinated with 1 Dose	Vaccinated with 2 Doses	Vaccinated with 3 Doses	Not Vaccinated
Post-COVID syndrome	13	61	51	37
No post-COVID syndrome	33	117	186	51

**Table 3:** Descriptive characteristics of SARS-CoV-2 infections in the study cohort.

SARS-CoV-2 Infection	Clinical Form	Number (%)
Severity of infection	Asymptomatic	48 (8.7%)
	Mild form, not hospitalized	438 (79.8%)
	Hospitalized, with no supplemental oxygen	48 (8.7%)
	Hospitalized with oxygen provided by a nasal cannula	7 (1.3%)
	Hospitalized with high-flow oxygen	7 (1.3%)
	Intubated	1 (0.2%)
Reinfection	1 episode	133 (24.2%)
	More than 2 episodes	47 (8.6%)
Reinfection severity	Worse the first time	85 (47.5%)
	Always the same severity	48 (26.8%)
	Worse the second time	34 (18.9%)
	Worse the third/fourth time	12 (6.7%)

**Table 4:** Persistent symptoms at different time intervals after the acute infection.

	Persistent Symptoms after the Acute Infection (*n*, %)			
Symptom	During the First 4 Weeks *n* = 549	After 3 Months *n* = 162	After 6 Months *n* = 129	After 12 Months *n* = 101
Persistent fever	223 (40.6%)	2 (0.4%)	1 (0.2%)	0
Anosmia/ageusia/dysgeusia	279 (50.8%)	47 (8.6%)	32 (5.8%)	18 (3.3%)
**Fatigue/asthenia**	**440 (80.1%)**	**93 (16.9%)**	**71 (12.9%)**	**48 (8.7%)**
Persistent headache	325 (59.2%)	47 (8.6%)	32 (5.8%)	20 (3.6%)
Dysphagia/odynophagia	240 (43.7%)	13 (2.4%)	10 (1.8%)	7 (1.3%)
Dyspnea	171 (31.1%)	43 (7.8%)	32 (5.8%)	16 (2.9%)
Myalgia/arthralgia	334 (60.8%)	53 (9.7%)	41 (7.5%)	29 (5.3%)
Persistent cough	266 (48.5%)	28 (5.1%)	17 (3.1%)	7 (1.3%)
Rhinorrhea/persistent sneezing/sore eyes	244 (44.4%)	29 (5.3%)	19 (3.5%)	11 (2%)
Adenopathies	81 (14.8%)	14 (2.6%)	12 (2.2%)	7 (1.3%)
Anorexia	147 (26.8%)	13 (2.4%)	9 (1.6%)	7 (1.3%)
Nausea/vomiting/gastro-intestinal disturbance	117 (21.3%)	19 (3.5%)	16 (2.9%)	7 (1.3%)
**Cognitive dysfunctions**	**190 (34.6%)**	**63 (11.5%)**	**49 (8.9%)**	**35 (6.4%)**
Anxiety/depression	144 (26.2%)	49 (8.9%)	35 (6.4%)	24 (4.4%)
Palpitations	171 (31.1%)	65 (11.8%)	50 (9.1%)	33 (6%)
**Sleep disturbances**	**199 (36.2%)**	**62 (11.3%)**	**53 (9.7%)**	**39 (7.1%)**

**Table 5:** Risk factors for post-COVID-19 symptomatology.

	Pearson/Fisher’s Test (*p*-Value)	OR	95% CI Min–Max	
Not being vaccinated	0.0047	2.046	1.245	3.363
Obesity	0.761	1.085	0.641	1.836
Presence of any comorbidity pre-infection	0.008	1.657	1.138	2.413
Reinfection	<0.001	2.405	1.642	3.523
Number of symptoms in the first 4 weeks >5	<0.001	4.243	2.809	6.411

**Table 6:** Logistic regression results of all statistically relevant possible risk factors in developing long COVID.

Tested Variable	ROC *p*-Value	Area Under the Curve	OR Logistic Regression (*p*-Value)	95% CI	
				Lower	Upper
Number of pre-infection comorbidities	0.009	0.571	3.637 (*p* = 0.012)	2.376	5.568
Reinfection episodes	<0.001	0.600	1.673 (*p* = 0.004)	1.118	2.502
Number of acute symptoms	<0.001	0.716	1.968 (*p* < 0.001)	1.149	3.371
Status—not vaccinated	0.087	0.546	1.824 (*p* = 0.014)	1.212	2.744

## Data Availability

The data presented in this study are available from the corresponding author due to privacy reasons upon request.

## Supplementary Materials

The following supporting information can be downloaded at: https://www.mdpi.com/article/10.3390/life14111519/s1.
