# Supplementary material for: Insights into the Risk Factors and Outcomes of Post-COVID-19 Syndrome—Results from a Retrospective, Cross-Sectional Study in Romania
Source: Life (Basel). 2024 Nov 20;14(11):1519. doi: 10.3390/life14111519 (PMC11596014; doi:10.3390/life14111519)
Supplement: Supplementary file 1 [file life-14-01519-s001.zip › life-3289877-supplementary.pdf]

Supplementary Materials-questionnaire

Consent for completion (18+ yo criteria)
Inclusion criteria question: Have you been infected with/positively tested for SARS-CoV-2? Yes/No

A. General data

Gender- Male/Female/Prefer not to say
Age-Single-answer question- Age category
Background- Rural/Urban
Level of education achieved- Single-choice answer
Occupation status before infection- Single-choice answer
Health-care worker at time of completion or in the past? Yes/No/Prefer not to disclose

B. General health status pre-infection and quality of life

Weight- answer in kilograms
Height- answer in centimetres
Smoker status- single-choice answer
Chronic conditions pre-infection- Multiple choice answer with open-ended possibility for other condition not listed-
Quality of life evaluation- pre-infection period- 3-step scale of impairment for 5 categories - mobility, self-care, usual activities, pain/discomfort, anxiety/depression

C. Vaccine status

Are you vaccinated against COVID-19? Yes/No (optional section based on answer to this question)
When was the first dose of the vaccine administered? Date: MM/DD/YY
When was the second dose of the vaccine administered? Date: MM/DD/YY
When was the booster dose of the vaccine administered? Date: MM/DD/YY
Which one of the following vaccines available in Romania were administered in your case? Single-choice answer for each dose administered
If the vaccine has been administered after the first COVID-19 infection, have you noticed an amelioration of your latent COVID-19 symptoms? Single-choice answer

D. SARS-CoV-2 infection information

When was the first time you were infected with SARS-CoV-2? MM/DD/YY
How were you diagnosed with COVID-19? - Antigen test, by a medical specialist, autodiagnosis based on recent contact with a positive subject
Did you require hospitalisation for your symptoms? No, I was asymptomatic/ I had mild symptoms/ I was admitted, but did not require supplemental oxygen/ I was admitted and required supplemental O2 by nasal cannula/ I required hospitalisation and required NIV/HFO/ I required mechanical ventilation/ I was hospitalised, but do not remember the level of ventilation I required
When hospitalised, did you receive any of the available antiviral medications in Romania? Yes/No/Do not remember
Have you had more than one bout of COVID-19? No/ Yes, I had a second episode of COVID-19/ Yes, I had multiple episodes
If you answered yes to the previous question, comparing the severity of the episodes you could state that: Single-choice answer

E. COVID-19 symptoms and their latency in time

Choosing the most severe episode of infection, at 4 weeks following the beginning of the symptomatology, did you consider yourself to be fully healed? Yes/ No, I still had symptoms/ No, but I had no more symptoms
If you answered “No[...]” to the last question, how could you describe the evolution of your symptoms over time? Single-choice answer
Please check each box for the symptoms you experienced during your COVID-19 episode and after, with their respective persistence in time periods. accordingly.- Multiple-choice table with Symptoms

listed as Rows and “At 4 weeks”/”After 2 months”/”After 3 months”/ “After 6 months”/ “After 12 months” as Columns

The remaining symptoms can be aggravated by- Multiple-choice answer and open-ended possibility for other unlisted aggravating factors

Have you experienced any other symptom that was not listed in the table above? Open-ended question

Have you required any further medical assistance in managing your persistent symptoms or ended up taking more time off work than the obligatory isolation period? Semi-structured multiple choice question and other

Did you experience an aggravation of your already existing chronic conditions after the COVID-19 infection? Yes/No and others

Were you diagnosed with a new chronic condition in the first 3 months post-COVID-19? Yes/No

If you answered “Yes” to the previous question, for which type of disease did you get a formal diagnosis on? Semi-structured, multiple-choice answer and others

F. Quality of life post-COVID-19

What is your current occupational status? Single-choice answer

Quality of life evaluation- post-infection period- 3-step scale of impairment for 5 categories - mobility, self-care, usual activities, pain/discomfort, anxiety/depression

Where 100% represents the state of your overall health before COVID-19, how could you rate your current overall health, post-infection?

For those willing and consenting to participate in further studies, please enter an email address for further communication (+added disclaimer on data storage and confidentiality)
